# Supplementary material for: Time-series transcriptome analysis identified differentially expressed genes in broiler chicken infected with mixed Eimeria species
Source: Front Genet. 2022 Aug 8;13:886781. doi: 10.3389/fgene.2022.886781 (PMC9393255; doi:10.3389/fgene.2022.886781)
Supplement: Supplementary file 2 [file DataSheet1.ZIP › 7dpi_KEGG.Gsea.1625115961950/KEGG_VIBRIO_CHOLERAE_INFECTION.html]

Details for gene set KEGG\_VIBRIO\_CHOLERAE\_INFECTION[GSEA]

|  || Dataset | TMM\_7dpi\_gct\_format\_7dpi\_gct\_format.Class\_4dpi.cls #PC\_versus\_NC.Class\_4dpi.cls #PC\_versus\_NC\_repos |
| Phenotype | Class\_4dpi.cls#PC\_versus\_NC\_repos |
| Upregulated in class | 1 |
| GeneSet | KEGG\_VIBRIO\_CHOLERAE\_INFECTION |
| Enrichment Score (ES) | 0.6168871 |
| Normalized Enrichment Score (NES) | 2.6627796 |
| Nominal p-value | 0.0 |
| FDR q-value | 0.0 |
| FWER p-Value | 0.0 |
Table: GSEA Results Summary

  

Fig 1: Enrichment plot: KEGG\_VIBRIO\_CHOLERAE\_INFECTION      
 Profile of the Running ES Score & Positions of GeneSet Members on the Rank Ordered List

  

| SYMBOL | TITLE | RANK IN GENE LIST | RANK METRIC SCORE | RUNNING ES | CORE ENRICHMENT || 1 | ATP6V1C2 | na | 4 | 2.128 | 0.0763 | Yes |
| 2 | ATP6V0A4 | na | 19 | 1.686 | 0.1359 | Yes |
| 3 | ATP6AP1 | na | 21 | 1.657 | 0.1955 | Yes |
| 4 | ATP6V0D2 | na | 24 | 1.636 | 0.2542 | Yes |
| 5 | ATP6V0C | na | 30 | 1.603 | 0.3115 | Yes |
| 6 | ATP6V1D | na | 46 | 1.458 | 0.3628 | Yes |
| 7 | ATP6V1E1 | na | 61 | 1.395 | 0.4119 | Yes |
| 8 | ATP6V0E1 | na | 72 | 1.354 | 0.4598 | Yes |
| 9 | ATP6V1H | na | 80 | 1.313 | 0.5065 | Yes |
| 10 | ATP6V0B | na | 100 | 1.252 | 0.5500 | Yes |
| 11 | ATP6V1G3 | na | 102 | 1.247 | 0.5949 | Yes |
| 12 | SEC61B | na | 410 | 0.813 | 0.5985 | Yes |
| 13 | KCNQ1 | na | 509 | 0.738 | 0.6169 | Yes |
| 14 | SEC61G | na | 960 | 0.535 | 0.5985 | No |
| 15 | SLC12A2 | na | 1482 | 0.385 | 0.5689 | No |
| 16 | ADCY9 | na | 2142 | 0.246 | 0.5227 | No |
| 17 | ATP6V1G1 | na | 2444 | 0.199 | 0.5047 | No |
| 18 | KDELR2 | na | 3177 | 0.104 | 0.4473 | No |
| 19 | ERO1A | na | 3322 | 0.084 | 0.4382 | No |
| 20 | KDELR3 | na | 3474 | 0.066 | 0.4280 | No |
| 21 | ARF1 | na | 3820 | 0.025 | 0.4001 | No |
| 22 | TCIRG1 | na | 3866 | 0.020 | 0.3970 | No |
| 23 | ATP6V1C1 | na | 4112 | -0.007 | 0.3768 | No |
| 24 | ACTB | na | 4316 | -0.032 | 0.3610 | No |
| 25 | ATP6V0A1 | na | 4522 | -0.052 | 0.3458 | No |
| 26 | SEC61A2 | na | 4576 | -0.058 | 0.3434 | No |
| 27 | ADCY3 | na | 5416 | -0.141 | 0.2784 | No |
| 28 | SEC61A1 | na | 5439 | -0.142 | 0.2817 | No |
| 29 | ATP6V0D1 | na | 5522 | -0.151 | 0.2803 | No |
| 30 | ATP6V1A | na | 5917 | -0.192 | 0.2543 | No |
| 31 | ATP6V1B2 | na | 5973 | -0.199 | 0.2568 | No |
| 32 | PLCG1 | na | 6241 | -0.228 | 0.2427 | No |
| 33 | ATP6V0E2 | na | 6836 | -0.290 | 0.2036 | No |
| 34 | PDIA4 | na | 7722 | -0.387 | 0.1435 | No |
| 35 | PRKX | na | 8092 | -0.434 | 0.1283 | No |
| 36 | GNAS | na | 8122 | -0.437 | 0.1416 | No |
| 37 | TJP2 | na | 8188 | -0.444 | 0.1522 | No |
| 38 | PRKCA | na | 8704 | -0.513 | 0.1276 | No |
| 39 | PRKACB | na | 9015 | -0.550 | 0.1216 | No |
| 40 | ATP6V0A2 | na | 9318 | -0.602 | 0.1180 | No |
| 41 | PRKCB | na | 9867 | -0.685 | 0.0969 | No |
| 42 | CFTR | na | 9974 | -0.707 | 0.1135 | No |
| 43 | TJP1 | na | 10016 | -0.715 | 0.1358 | No |
| 44 | PLCG2 | na | 10697 | -0.853 | 0.1097 | No |
Table: GSEA details [plain text format]

  

Fig 2: KEGG\_VIBRIO\_CHOLERAE\_INFECTION      
 Blue-Pink O' Gram in the Space of the Analyzed GeneSet

  

Fig 3: KEGG\_VIBRIO\_CHOLERAE\_INFECTION: Random ES distribution      
 Gene set null distribution of ES for **KEGG\_VIBRIO\_CHOLERAE\_INFECTION**
